# Automatic Detection of Missing Access Points in Indoor Positioning System †

**DOI:** 10.3390/s18113595

**Published:** 2018-10-23

**Authors:** Rafał Górak, Marcin Luckner

**Affiliations:** Faculty of Mathematics and Information Science, Warsaw University of Technology, Koszykowa 75 street, 00-662 Warsaw, Poland

**Keywords:** indoor localisation system, fingerprinting, system deployment and maintenance

## Abstract

The paper presents a Wi-Fi-based indoor localisation system. It consists of two main parts, the localisation model and an Access Points (APs) detection module. The system uses a received signal strength (RSS) gathered by multiple mobile terminals to detect which AP should be included in the localisation model and whether the model needs to be updated (rebuilt). The rebuilding of the localisation model prevents the localisation system from a significant loss of accuracy. The proposed automatic detection of missing APs has a universal character and it can be applied to any Wi-Fi localisation model which was created using the fingerprinting method. The paper considers the localisation model based on the Random Forest algorithm. The system was tested on data collected inside a multi-floor academic building. The proposed implementation reduced the mean horizontal error by 5.5 m and the classification error for the floor’s prediction by 0.26 in case of a serious malfunction of a Wi-Fi infrastructure. Several simulations were performed, taking into account different occupancy scenarios as well as different numbers of missing APs. The simulations proved that the system correctly detects missing and present APs in the Wi-Fi infrastructure.

## 1. Introduction

Localisation services are part of our daily routine. While services based on Global Positioning System (GPS) provide sufficient accuracy when positioning in the outdoor environment, the issue of providing an accurate indoor position is much more complicated. First, all GPS solutions fail inside buildings. Second, a higher level of accuracy is required indoors than outdoors. This is because even a relatively small one-meter localisation error in the indoor environment may mean that the device being localised is in a different room or on a different floor. Hence, different solutions have to be applied. Usually, the most accurate localisation systems use different indoor localisation solutions or require the installation of particular devices inside the building that will enhance the localisation but at additional costs.

This paper proposes a localisation solution that is based on received signal strength (RSS) from various access points (APs) of the Wi-Fi infrastructure inside the building. A Wi-Fi localisation system using RSS is one of the most popular due to its low cost and accessibility as the measurements can be performed on almost every mobile device with a Wi-Fi module. Additionally, the growing popularity of different mobile inertial sensors like gyroscopes or accelerometers enables creation of pedestrian reckoning systems (PDR). Hence, quite often the localisation solution uses a fusion of PDR- and RSS-based localisation obtaining very good accuracy [1,2,3,4].

By measuring Wi-Fi signal strengths from multiple APs in various locations we can create a map of fingerprints. This off-line phase is called fingerprinting. In the localisation process, one can find their position by comparing a vector of the current signal strengths to the created map of the fingerprints. Although the idea is straightforward, the fact that RSS suffers from significant fluctuations due to the complex indoor radio propagation conditions makes the problem of creating an accurate localisation solution very challenging.

We present a solution that can be used for developing existing localisation solutions using various localisation systems where an RSS-based localisation model is one of them. The solution described in this paper has the following advantages. First of all, the most critical component of the localisation solution is the system detecting missing APs. Some APs that were observed during the offline phase of model creation (fingerprinting phase) may disappear from the building due to breakdown or removal. Most probably this will affect the accuracy of the localisation model. We solved this problem by incorporating in the localisation model a component responsible for detecting missing APs. Another essential feature of our localisation model is that it provides the localisation based only on a single reading not taking into account the readings taken previously. Although it is not a dead reckoning solution, it can be a base model to create solutions by using previously determined positions in the localisation process (see for example [5,6]).

The method proposed here makes extensive use of the Random Forest algorithm [7]. The main feature of this solution is that it is very time efficient in the process of model creation as well as when the model is applied for providing a localisation. It should be mentioned here that quite often the solution uses nearest neighbours algorithm at least as the reference algorithm (see [8,9,10,11,12]). Although the method can be entirely accurate when it is applied to larger, possibly multi-floor buildings, it is not going to be very time efficient. The reason for that is the fact that the radio map of fingerprints collected in the offline phase is a big data set whenever we have a large area to cover. Hence, the nearest neighbour algorithms require a comparison with all the elements of the reference data set, and thus are time expensive.

**Figure 1:**
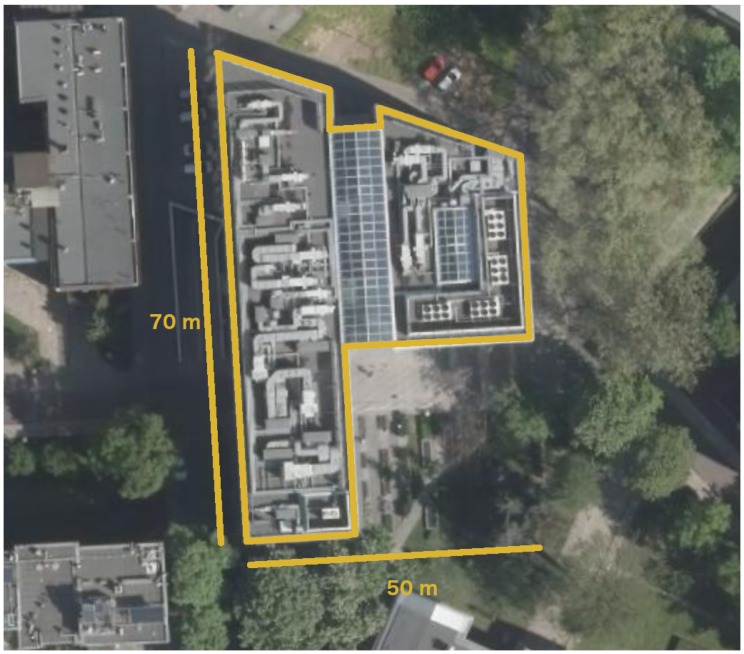
Faculty of Mathematics and Information Science (MIS) of Warsaw University of Technology.

Finally, it has to be emphasised that we tested our model inside of the big recently built multi-floor building of Faculty of Mathematics and Information Science (MIS) of the Warsaw University of Technology. The building is built mostly of glass, steel and concrete. As one can see in Figure 1, the building is also of irregular shape. As a consequence, we tested our solution in a diverse environment from the perspective of signal propagation.

From that perspective, the results presented in this paper differ from most of the localisation solutions presented in various other articles, as they usually cover only one floor and are limited to a small area. This article is an extension of two conference papers [13,14].

The remaining part of the paper is organised as follows: in Section 2 related work is discussed. Section 3 presents basic facts about the analysed data and localisation methods. Data sets of fingerprints collected in the MIS building are described and discussed in Section 4. Section 5 presents the proposed automatic indoor positioning system. The testing procedure, which simulates real-life application of the proposed localisation system, is described in Section 6 as well as the obtained results. Section 7 concludes the paper.

## 2. Related Work

Various approaches to Wi-Fi-based localisation have been presented in several works. A survey of indoor positioning systems for personal wireless networks can be found in work [15]. Recent advances in Wi-Fi fingerprint localisation were summarised in [16].

One of the main issues of Wi-Fi-based localisation is the variation of received signals. Several works have proposed modification or normalisation of the obtained signal to reduce the problem of variation. Work [17] proposed calculating discriminative components for network learning. The nonlinear relationship between RSS and the position was then accurately constructed by incrementally inserting the discriminative components and recursively updating the weightings in the network until no further improvement was required. Work [18] provided an Adaptive Fingerprint Update (AFU) to cope with the environmental changes. AFU used a novel adaptive fingerprint update technique to adjust the algorithm according to scenario variations based on Kriging interpolation.

Work [19] proposed a TKL-WinSMS strategy, which realised an adaptive indoor localisation in dynamic indoor environments. A Wi-Fi-based Non-intrusive Sensing and Monitoring System (WinSMS) enabled Wi-Fi routers as online reference points by extracting real-time RSS readings among them. These online data and labelled source data from the offline calibrated radio map were combined with the RSS readings from target mobile devices as unlabelled target data, to design a localisation model using an emerging transfer learning algorithm, namely transfer kernel learning (TKL). It was able to learn a domain invariant kernel by directly matching the source and target distributions in the reproducing kernel Hilbert space instead of the raw noise signal space. It improved the referential Support Vector Regression results by 1.18 m on 15 testing points.

A real-time adaptation to changes in Wi-Fi infrastructure is proposed in work [20]. The CRIL system coupled the inertial navigation system (INS) and the received signal strength indicator (RSSI) to obtain better localisation performance. The system used the results from RSSI and INS and updated the channel model in the RSSI in real time. The system could track the dynamic channel model to provide more accurate localisation results. The proposed method reduces the drift error to less than one meter on a 35 m path. An alternative self-calibrating and self-adaptive model was proposed in [6], which merges two models, a free space path loss model and a propagation model. This self-calibrating procedure utilises one propagation model to infer parameters of the space and the other to simulate the propagation of the signal. The authors used historical points for localisation improvement and stressed the issue of not correctly detected or missing APs.

Work [21] proposed using low-cost off-the-shelf two-way time-of-arrival (TW-ToA) ranging devices to perform localisation. The main idea of their approach was that even without explicit knowledge of the RSS values, use could be made of the connectivity information. The approach was based on non-linear regression analysis where the missing observations were treated as Missing Not at Random (MNAR). Similar ideas were proposed in [22,23]. However, the support for this technology depends on a used chipset. In indoor localisation, high-level nanosecond time measuring precision is needed when the standard 802.11 synchronises clocks with microsecond precision. Therefore, the technology is hard for broad implementation on commonly used mobile phones [24].

Another approach is proposed in [25,26] where a map created during the fingerprinting process is recovered in place of the missing signals. Work [27] discussed—on the laboratory testbed—how to recover missing APs’ RSS if the radio map covers all measurement points for all APs. The proposed technique cannot be used in the whole building. However, we discuss this approach in depth and compare it on the subset of collected data in Section 6.3.

The tests in our work were performed in a multi-floor building, while most of the cited works are focused on horizontal localisation only. The presented solution is based on preliminary work [13]. Other analyses for the building can be found in [28,29,30]. However, these works are focused on the localisation algorithms and their results cannot be directly compared with our results.

## 3. Preliminaries and Notation

This section will describe the already mentioned fingerprinting method and the precise formulation of the localisation.

### 3.1. Fingerprinting

The fingerprinting approach for indoor localisation is to build a model based on a data set of training fingerprints, which given an RSS vector will predict its position. Let us describe formally the fingerprinting concept following the notation used in [13]:

**Definition** **1.**

*(i)*  AP is the set of all APs used for the localisation model enumerated by the consecutive natural numbers (1,2,3…).
*(ii)*  $F=R^{2}\times Z\times R\times R^{n}$ is the space of fingerprints where n=♯AP.
  For f∈F
  *(a)*  the coordinates $f_{1}\left[m\right],f_{2}\left[m\right]$ denote the horizontal location of the measurement point;
  *(b)*  the coordinate $f_{3}\in Z$ denotes the floor where the measurement point is placed;
  *(c)*  $f_{4}\left[s\right]$ is the time of the measurement;
  *(d)*  $f_{k}\left[dBm\right]$, where 4<k≤n+4, is the RSS from the kth source from AP. If there is no signal from the kth AP then $f_{k+4}=\emptyset$, where *∅* is a special unique value.
*(iii)*  A set of fingerprints S⊂F defines a measurement series. Usually S is collected during one or a few consecutive days on the same set of measurement points in a particular building.
*(iv)*  $L=\left(L_{x},L_{y},L_{f}\right):F\mapsto R^{2}\times Z$ is the projection onto the first three coordinates of the set F and $\pi :F\mapsto R^{n}$ is the projection onto the last n coordinates. In other words L(f) provides us with the location of a fingerprint f while π(f) is the RSS vector associated with f (fingerprint).
*(v)*  For $v=\left(v_{1},\ldots ,v_{n}\right)\in R^{n}$ we denote $supp\left(v\right)=\left\{k:\;v_{k}\neq \emptyset \right\}$ which is the set of visible APs for the fingerprint v.

### 3.2. Localisation Model

Let us precisely formulate the localisation problem that is solved in the following part of this article.

**Problem** **1.** *Based on a measurement series $S_{L}$(learning series) construct a location model. The localisation model is a function $\hat{L}:R^{n}\mapsto R^{2}\times Z$ such that given an RSS vector $v\in R^{n}$, $\hat{L}\left(v\right)$ predicts a localisation where the measurement v was taken.*

One can see that in such a formulation of the localisation problem we do not take into account the previous RSS readings if there were any. The localisation is based on just one RSS reading from multiple APs.

In order to evaluate the model we introduce the following standard measures of accuracy:

**Definition** **2.** Let $S_{T}$ (testing series) be a measurement series and $\hat{L}$ a localisation model. For an element $s\in S_{T}$ we introduce the following notions:

*horizontal error*

$$E_{h}\left(\hat{L},s\right)=\sqrt{{\left(\hat{x}-x\right)}^{2}+{\left(\hat{y}-y\right)}^{2}}$$

*and the floor error:*

$$E_{f}\left(\hat{L},s\right)=\left|\hat{f}-f\right|,$$

*where $\hat{L}\left(\pi \left(s\right)\right)=\left(\hat{x},\hat{y},\hat{f}\right)$ and L(s)=(x,y,f). In other words (x,y,f) is the truth position of fingerprint s while $\left(\hat{x},\hat{y},\hat{f}\right)$ is the predicted position based on RSS vector π(s).*

**Definition** **3.** *For a testing series $S_{T}$ and the localisation model $\hat{L}$ let us define:*

*(i)*  *Mean horizontal error*
  $$\mu E_{h}\left(\hat{L},S_{T}\right)=mean\left\{E_{h}\left(\hat{L},s\right):\;s\in S\right\};$$
*(ii)*  *Classification error for floor’s prediction*
  $$\varepsilon_{f}\left(\hat{L},S_{T}\right)=\frac{♯\{s\in S_{T}:\;E_{f}\left(\hat{L},s\right)\neq 0\}}{♯S_{T}};$$

*When it is clear from the context which model is tested and what testing series is used, the symbol of the model and testing series will be omited, i.e.,: $\mu E_{h}=\mu E_{h}\left(\hat{L},S_{T}\right)$, $\varepsilon_{f}=\varepsilon_{f}\left(\hat{L},S_{T}\right)$.*

Obviously the main goal in the localisation problem is to make $\mu E_{h}$ and $\varepsilon_{f}$ as small as possible.

## 4. Data Sets Explained

### 4.1. Fingerprints

The analysis will be conducted on data collected in a multi-floor building. Data were collected in publicly accessible areas of the building. The building is of irregular shape and their outer dimensions can be read from Figure 1.

The data were collected in two independent series. The first series $S_{L}$ was used as the learning set, the second one $S_{T}$ as the testing set. The learning set contains data collected between 18 August 2014 and 22 August 2014. Data gathered between 25 August 2014 and 29 August 2014 created the testing set.

**Figure 2:**
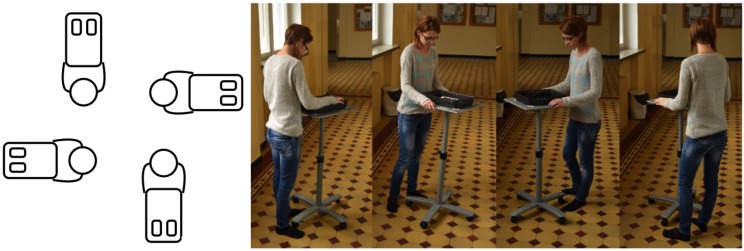
Measurement procedure.

The data were collected using the Android application created for the localisation system [31]. The application worked on Android OS 2.1 (or newer) running at three different models of mobile phones: HTC One, LG Nexus4, and Sony Xperia. During the measurements the phones were transported and rotated on a trolley (see Figure 2).

**Figure 3:**
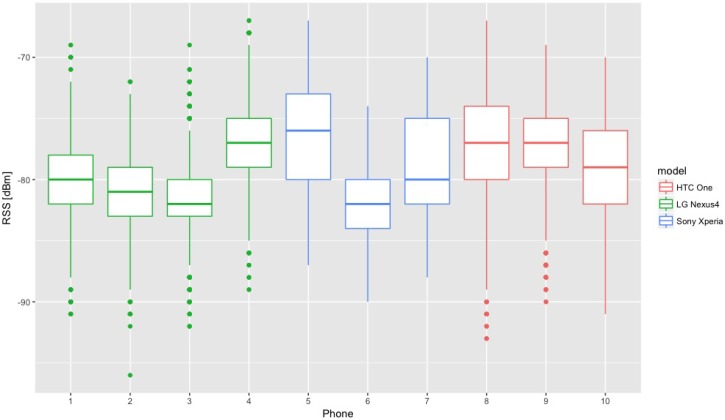
Difference in RSS for various phones and models used in data gathering.

Figure 3 shows how RSS from the same A varies for different phones and models used in the data gathering. The presented test was made using ten different phones simultaneously collecting data and laying on the same trolley. The data were collected from one AP over 90 min. All phones registered the similar number of measurements that was about 2400.

The differences between RRS are small. The highest difference between the means is 2 dBm and 6 dBm for the models and the individual phones respectively.

In each of two series, the location fingerprints create mostly a 1.5 × 1.5 m grid. Only when it was impossible due to the building’s structure—walls or different obstacles—the grid was slightly sparser. Both series cover the same area of the building and the points of the grid corresponding to $S_{L}$ and $S_{T}$ are shifted by 0.75 m in each direction, so they do not intersect. There were 40 fingerprints taken at every measurement point. The measurements have been done in four directions parallel to the building axes. The purpose of it is to take into account RF power absorption of a human body. This way, the collected data sets $S_{L}$ and $S_{T}$ contain measurements that consider different positions of a human body with respect to the terminal and AP.

Table 1 shows the number of fingerprints in each series for every building. Hence the number of measurement points (elements of the grid) is around 1100–1200 for each series.

Finally, it should be explained that we set ∅=−117 [dBm]. It is the value that is below the minimum value that can be reported by the mobile devices used in the process of gathering fingerprints. From the perspective of the method (Random Forests) that will be used for creating localisation models it can be any value below the minimum signal strength that can be measured.

### 4.2. Graph

For simulating real-life situations to test our solution we created a graph $G_{T}$ whose vertices are the points where fingerprints were taken in the testing series $S_{T}$. Let us denote this set by $V_{T}$. As it was mentioned in the previous section, the measurement points are distributed in a square grid. Therefore, each vertex can be connected to eight nearest measurement points by edges.

Each of the edges is one of three types:

free—the edge does not intersect any obstacle and there is a free passage along the edge.
door—the edge intersects the door
wall—all other cases. That includes: the edge intersects a wall or any other unmovable obstacle.

**Figure 4:**
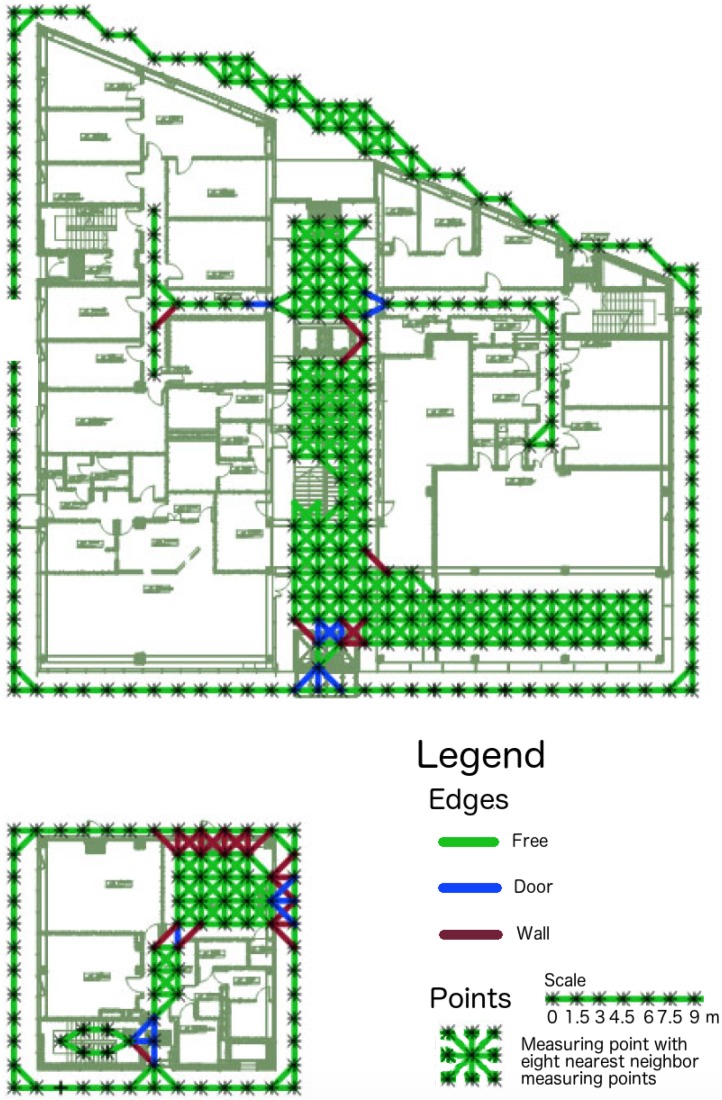
The plan of the first floor of the MIS building with a marked graph.

Figure 4 presents a part of this graph on the first floor of the MIS building. This part of the graph contains two separate parts but for the whole building the graph is connected.

During testing procedure we will consider graph that consists of edges of type free and door only. We denote this set of edges by $E_{T}$. We do not include the edges of type wall as we want to simulate movements of people inside the building while testing the proposed localisation solution. The similar graph $G_{L}$ is created for learning data set $S_{L}$.

## 5. Self-Correcting Localisation System

**Figure 5:**
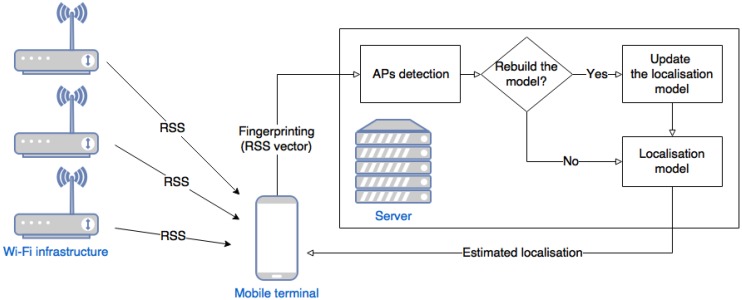
Localisation system.

We propose a localisation system that will automatically detect missing APs and appropriately correct the localisation model. The system also detects APs that are present, which enables us to include in the model APs that were previously removed. The whole system is presented in Figure 5.

A mobile device collects information on RSS from all APs in the range. The device does not have to be connected to any of these APs. The mobile phone sends the collected information to a server with a localisation service using the localisation model. Initially, the model learns on selected signal sources. The selection removes incidentally collected data such as unstable signals from other buildings or mobile hot-spots. The selection mechanisms are described in Section 5.1.

As the input of the model we take a collection of sets ${\left\{{\mathrm{RSS}}_{s}\left(N\right)\right\}}_{s\in \mathrm{AP}}$ and the set S⊂AP of APs that are used for predicting the localisation by ${\hat{L}}_{RF}$. Next, Algorithm 1 returns us sets $S_{present}$ and $S_{missing}$ that allow us to replace the set *S* by $S=\left(S\cup S_{present}\right)\setminus S_{missing}$ and rebuild the model using features only from the new set *S* when creating ${\hat{L}}_{RF}$, as is described in Section 5.2. When the set *S* is unchanged, we do not update the model. Details are given in Section 5.3.

The model estimates the current location and returns the estimated location to the mobile device. The performance tests of the system are presented in Section 5.4.

### 5.1. Selection of Initial Signal Sources

For the creation of an efficiently working self-correcting localisation system the number of observed APs should be limited. For example for measurements in the MIS building 570 unique APs were observed. Analysis and scanning signals from all sources is very time consuming. Therefore, we propose a method of selection the most critical APs using their importance.

#### 5.1.1. Calculation of Importance

At each measurement point, a fingerprint vector *F* was created containing information on signal strength measures for each AP. Usually, at least several APs were not registered at the given measurement point. The measures for these APs are labelled with the special unique value ∅. The rest of the measures are given in decibel-milliwatt (dBm) and registered as negative numbers. In the subsequent calculations, we assumed that ∅ is a negative number smaller than the smallest observed signal.

To estimate the importance of APs a method built in the CART classification trees is used. The feature importance is calculated for a split defined by the given feature. In the classification task, the Gini coefficient is used instead to estimate how the data space in the node is divided among classes [32]. In each node *m* of tree *T* the Gini coefficient is calculated:

$I\left(m\right)=\sum_{i=1}^{g}f_{i}\left(1-f_{i}\right)=\sum_{i=1}^{g}\left(f_{i}-{f_{i}}^{2}\right)=\sum_{i=1}^{g}f_{i}-\sum_{i=1}^{g}{f_{i}}^{2}=1-\sum_{i=1}^{g}{f_{i}}^{2}$, where *g* is the number of classes in the classification task.

The coefficient for the node *m* is calculated using a subset of data $S_{m}\subset S$. The set includes observations from the observation space defined by conditions in node *m*. Moreover $f_{i}=\frac{n_{m_{i}}}{n_{m}},i=1,\ldots ,g$, where $n_{m}=card\left(S_{m}\right)$ is the number of records assigned to node *m*, and $n_{m_{i}}$ is a number of the observations $x_{k}\in S_{m}:y_{k}=i$ that belong to class *i*.

The method CART defines the measure of variation for node *m* of tree *T* as $Q_{m}\left(T\right)=I_{G}\left(m\right)$. For a split in the tree that creates two children $m_{L}$ and $m_{R}$ of the node *m* we can calculate the variation obtained by the children as $\frac{n_{m_{L}}}{n_{m}}Qm_{L}\left(T\right)+\frac{n_{m_{R}}}{n_{m}}Qm_{R}\left(T\right)$. Therefore, the quality of the split is given by the change of the variation

$\Delta Q_{m,m_{L},m_{R}}=Q_{m}\left(T\right)-\left(\frac{n_{m_{L}}}{n_{m}}Qm_{L}\left(T\right)+\frac{n_{m_{R}}}{n_{m}}Qm_{R}\left(T\right)\right)$

Our aim is the maximisation of the difference $\Delta Q_{m,m_{L},m_{R}}$ to obtain a small variation in nodes $m_{L}$ and $m_{R}$.

For a random forest, the used function computes estimates of predictor importance for all weak learners [7]. For every tree and for splits on every feature used in the recognition process the changes are summarised. Next, the sum is divided by the number of branch nodes. In the regression tree, the difference between Mean Squared Error (MSE) for the parent node and the total MSE for the two children is calculated instead.

The importance of AP s∈AP is calculated on the basis of the importance calculated in the splits made on the base of the signal received from this AP.

For every AP s∈AP the importance is normalised to the range [0,1] using the following formula

(1) $${\bar{I}}_{s}=\frac{I_{s}}{\max_{a\in \mathrm{AP}}\left(I_{a}\right)}.$$

In the normalised importance, zero is representing the smallest possible importance and one the most significant importance.

#### 5.1.2. Selection of Learning Subset

We can distinguish two types of Wi-Fi APs for a learning series $S_{L}$. The first group $I_{L}\subset S_{L}$ belongs to the well-known building Wi-Fi infrastructure and mostly the building manager can diagnose their status. The second group $O_{L}\subset S_{L}$ contains Wi-Fi APs that do not belong to the infrastructure. This group—the outsiders—vary much more and include observed stationary APs from the neighbourhood as well as mobile APs.

**Figure 6:**
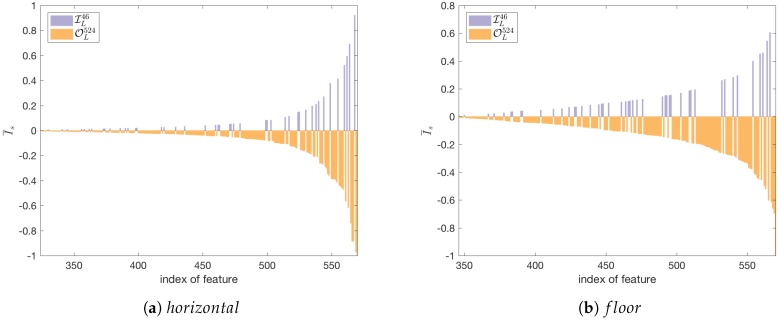
The importance of Wi-Fi APs calculated separately for horizontal localisation (**a**) and floor detection (**b**).

In the MIS building, we observed 46 APs from the infrastructure. The rest of them—over five hundred—were outsiders. It is safer to limit the localisation system to the APs from the infrastructure. On the other hand, the remaining APs can bring additional knowledge to the localisation system. Using the importance *I* we estimated the influence of the infrastructure on the localisation task. Figure 6 presents feature importance calculated for the various APs and importance calculated for the horizontal localisation task and the floor detection. In the case of the horizontal localisation, the importance was calculated as the average of the importance for the *x* and *y* coordinates.

The chart was limited to the APs with importance greater or equal 0.1. The APs from the infrastructure are marked as blue. The outsiders are marked with red. This shows how signals from the outsiders are important for the created localisation model. We also see that the number of important APs—with importance over 0.1—is greater for the horizontal localisation task (Figure 6a) than for the floor detection task (Figure 6b).

Let us order the observed APs in the learning set $S_{L}$ according to the importance $\bar{I}$. Let us define three subsets $I_{L}^{n}$,$O_{L}^{n}$,$S_{L}^{n}$ that consist of signals from the *n* APs with the highest importance. The first set included only the APs from the infrastructure. The second set consists of the APs that do not belong to the infrastructure. The last set is the union of both previous sets and contains all APs.

**Figure 7:**
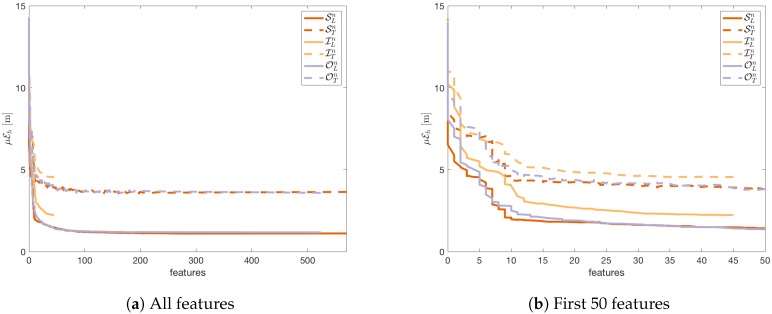
The dependence between the number of features and the obtained horizontal mean error $\mu E_{h}$.

Figure 7 shows how the reduction of the number of features by cutting off the APs with the lowest importance $\bar{I}$ changes the obtained $\mu E_{h}$ error. A comparison was made for three sets of features $I_{L}^{n}$,$O_{L}^{n}$,$S_{L}^{n}$. For each set of the features, random forests were created to predict positions defined by the fingerprints. The obtained $\mu E_{h}$ error was also calculated for the testing sets $I_{T}^{n}$,$O_{T}^{n}$,$S_{T}^{n}$. Similar tests were performed to show the influence of the number of features on the $\varepsilon_{f}$ error. The results are shown in Figure 7.

**Figure 8:**
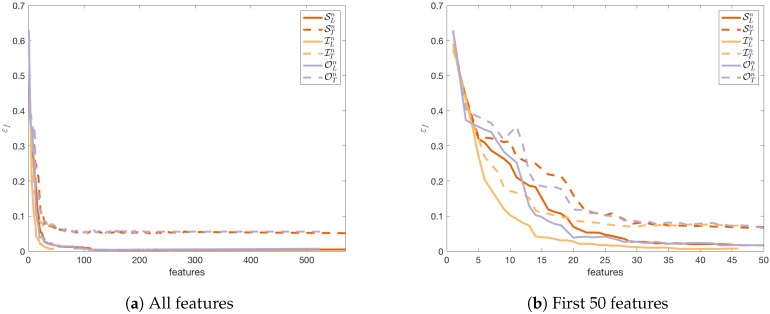
The dependence between the number of features and the obtained classification error for the floor’s predictions $\varepsilon_{f}$.

Figure 7a and Figure 8a show that we can reduce errors $\mu E_{h}$ and $\varepsilon_{f}$ by increasing the number of features considered in the localisation task. However, the localisation system will be less efficient when the number of features is very high.

For both errors, the characteristic of the curve for the learning sets and the testing sets are similar to each other. Therefore, we can assume that the reduction of the number of features will bring similar results for the testing set as for the learning set and the localisation error will not increase after the reduction of the number of features in comparison to the result obtained for the full set of features.

Some interesting observations can be made when we compare the obtained results on various datasets—the APs from the infrastructure $I_{T}^{n}$, the outsiders $O_{T}^{n}$, and the set of all features $S_{T}^{n}$—for the same number of features.

For the horizontal detection (Figure 7b) the results obtained on the learning sets using all the 46 APs from the infrastructure are worse than the results obtained on the other sets of APs. Meanwhile, the results obtained on the testing set for 46 features are nearly the same regardless of the used set of features. However, for the floor detection (Figure 8b) the observations are different.

First, the infrastructure APs give the best results on the learning set although the results are very similar. Second, the results obtained on the testing set for the APs from the infrastructure are noticeably worse than the results obtained on the other sets. Therefore, we can conclude that it is better to include the outsiders in the localisation system, but the selection of the outsiders should be controlled because the results obtained in this case can be slightly worse than in the case of the horizontal localisation.

In the discussed case the number of the APs from the building infrastructure is relatively small. Therefore, we face the issue of the selection of the cut-off threshold when the set of outsiders is taken into consideration in the localisation task with or without the APs from the infrastructure.

The selection of the cut-off point can be taken from observation of the error reduction in the learning set according to the number of used features in the localisation task. A first approach selects the number of features that gives the minimal error on the learning set according to the following formulae

(2) $$\begin{array}{cc} m_{h} & =\underset{n\leq N}{arg min}\left(\mu E_{h}\left(\hat{L},S_{L}^{n}\right)\right), \end{array}$$

(3) $$\begin{array}{cc} m_{f} & =\underset{n\leq N}{arg min}\left(\varepsilon_{f}\left(\hat{L},S_{L}^{n}\right)\right). \end{array}$$

However, for the descending error functions $\mu E_{h}$ and $\varepsilon_{f}$ (Figure 7 and Figure 8 respectively) is expected that values $m_{h}$ and $m_{f}$ will be close to the maximal number of the features *N*. The alternative approach limits the set of features to the subset that exceeds the error close to the best result. The acceptable change is defined by thresholds $\theta_{f}$ and $\theta_{h}$. According to the obtained quality, the coefficients can be fixed at 0.5 percent and 5 centimetres respectively. The cut-off is calculated using the following formulae

(4) $$\begin{array}{cc} n_{h} & =\min\{n:\mu E_{h}\left(\hat{L},S_{L}^{n}\right)-\mu E_{h}\left(\hat{L},S_{L}^{m_{h}}\right)<\theta_{h}\wedge n\leq m_{h}\}, \end{array}$$

(5) $$\begin{array}{cc} n_{f} & =\min\{n:\varepsilon_{f}\left(\hat{L},S_{L}^{n}\right)-\varepsilon_{f}\left(\hat{L},S_{L}^{m_{f}}\right)<\theta_{f}\wedge n\leq m_{f}\}. \end{array}$$

To decide which set of features should be used as the final one the errors $\mu E_{h}$ and $\varepsilon_{f}$ obtained on the sets must be compared.

**Figure 9:**
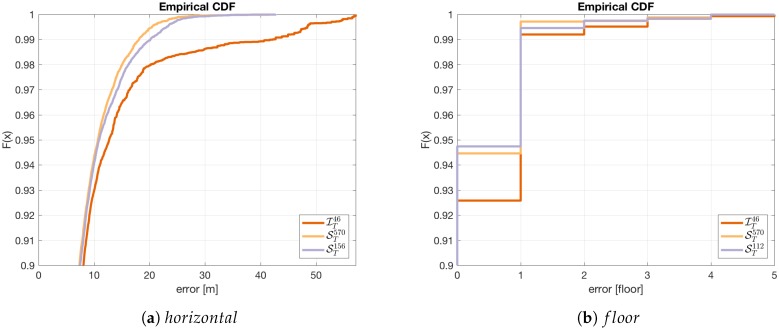
The comparison of the errors $\mu E_{h}$ and $\varepsilon_{f}$ obtained for localisation system working on three different sets of features.

Figure 9 presents the results obtained for the testing sets $S_{T}^{N}$,$S_{T}^{n_{f}}$, $S_{T}^{n_{h}}$, and $I_{T}^{46}$. The first set $S_{T}^{N}$ includes all 570 APs from the infrastructure and outsiders. The rest of the sets had a reduced number of features.

The sets $S_{T}^{n_{f}}$, $S_{T}^{n_{h}}$ contains signals from all kinds of APs. However, the number of signals was reduced to the most important APs using Formulae (4) and (5). The obtained number of features is $n_{h}=156$ for the horizontal localisation task and $n_{f}=112$ the for the floor detection task. The last set $I_{T}^{46}$ consists of all 46 APs from the infrastructure.

Figure 9 compares the results obtained by the discussed sets of the features using the empirical distribution function. Because of the high similarity of the plots the chart starts from 0.9.

In the case of the horizontal detection task (Figure 9a), we observe the differences between the errors obtained for various sets of features. The best result was obtained on the set containing all features. However, we can limit the number of observed APs to 27 percent using the reduced set of features. This is important for future system maintenance. It should be stressed that the differences between the results can be observed mostly for gross errors. A different situation can be observed for floor recognition (Figure 9b). The results vary mostly by the smallest error of one floor. The gross errors are on a very similar level.

### 5.2. Building Localisation Model

The main part of the localisation system is the localisation model as described in Section 3.2 in Problem 1. To construct the localisation model ${\hat{L}}_{RF}:R^{n}\mapsto R^{2}\times Z$ the Random Forest algorithm is applied as presented in [7]. It will be one of the main parts of the localisation solution.

First we create estimators ${\hat{L}}_{x}$, ${\hat{L}}_{y}$ and a classifier ${\hat{L}}_{f}$ by applying Random Forest algorithm where the training set is $\pi (S_{L})$ with responses $L_{x}\left(S_{L}\right)$, $L_{y}\left(S_{L}\right)$ and $L_{f}\left(S_{L}\right)$, respectively. For creating ${\hat{L}}_{x}$ and ${\hat{L}}_{y}$ regression trees are grown and for ${\hat{L}}_{f}$ the decision trees are grown. The selected number of grown trees is 30 as the analysis based on $S_{L}$ suggests that growing more trees does not improve accuracy.

Hence the localisation model is defined:

$${\hat{L}}_{RF}\left(v\right)=\left({\hat{L}}_{x}\left(v\right),{\hat{L}}_{y}\left(v\right),{\hat{L}}_{f}\left(v\right)\right).$$

For $v_{0}=\left(\emptyset ,\emptyset ,\ldots ,\emptyset \right)$ we set ${\hat{L}}_{RF}\left(v_{0}\right)=NaN$. However, it appears that the AP coverage is such that $v_{0}\notin \pi \left(S_{L}\right)\cup \pi \left(S_{T}\right)$ for MIS building.

It was already mentioned in the introduction that the central part of the proposed localisation system is detection of missing AP. A missing AP is one that was observed during the creation of the learning set but was damaged, turned off, or absent in the testing set.

To present how missing and undetected APs may influence the accuracy of the localisation model we remove APs in the testing data set $S_{T}$ and see how it influences different accuracy measures. The removed APs were selected using importance *I* (see Equation (1)). The most important APs were removed first to cause the biggest loss in accuracy.

Let us look at Table 2, which presents the decrease in accuracy when removing up to 10 APs. We can notice a major reduction of $\varepsilon_{f}$ even if only 2 APs are removed. When a missing AP is detected, we can update our model to perform almost as well as before the removal.

Table 2 shows that before a rebuilding action, the localisation algorithm increases $\mu E_{h}$ to over 7 m and $\varepsilon_{f}$ drops by 0.3 in case of an extensive malfunction. Meanwhile, $\mu E_{h}$ increases by less than 2 m and $\varepsilon_{f}$ drops only by 0.04 for the rebuild model.

### 5.3. Detection of Infrastructure Malfunction

The main part of the localisation solution presented in this paper is the system detecting missing APs. Section 5.2 shows how the accuracy may be improved when the localisation model ${\hat{L}}_{RF}$ is rebuilt. However, it cannot be made without a previous detection of the missing APs.

In order to build a detection system, for every AP s∈AP the predictor ${\hat{f}}_{s}:R^{n-1}\mapsto \left\{0,1\right\}$ is created. Based on the readings of the signal strength from all the remaining n−1 APs, ${\hat{f}}_{s}$ predicts whether there is a signal from APs *s*. The predictor ${\hat{f}}_{s}$ is created using the Random Forest algorithm. The number of trees is 20. In order for the system to detect missing APs, for every AP s∈AP, a collection ${\mathrm{RSS}}_{s}\left(N\right)$ of the last *N* readings is gathered for which the predictor ${\hat{f}}_{s}$ predicts that there should be a signal from AP *s* i.e., all readings $v\in R^{n}$ for which ${\hat{f}}_{s}\left({\overset{ˇ}{v}}^{s}\right)=1$ where ${\overset{ˇ}{v}}^{s}\in R^{n-1}$ denotes a vector with the *s* coordinate missing in *v*. If there are fewer than *N* readings for which ${\hat{f}}_{s}\left({\overset{ˇ}{v}}^{s}\right)=1$, then ${\mathrm{RSS}}_{s}\left(N\right)=\emptyset$. Algorithm 1 returns two sets of APs $S_{present}$ and $S_{missing}$. The set $S_{present}$ contains all the APs that were detected and classified as present. The set $S_{missing}$ contains all the APs that were classified as missing and parameter $p_{present}$ is a threshold parameter.

**Algorithm 1:** DetectAP algorithm

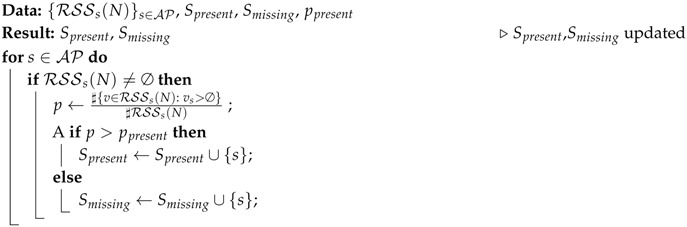



We arbitrarily set parameters N=100 and $p_{present}=0.1$. Let us recall that the terminal collects a single fingerprint every 0.5–1.5 s. Then the RSS vector is sent to the server. Hence the higher the number of terminals inside the building, the less the time needed to collect the last 100 readings where the signal from a given AP is expected. However, we believe that the parameter *N* should be tuned when the localisation system is working in a real-life situation, as it depends on the occupancy of the particular building. Concerning the parameter $p_{present}$, the most natural value would be $p_{present}=0$. This means that whenever we observe a signal from AP *s* at least once among the last *N* readings (where the signal from *s* is expected), then we treat it as present. Otherwise, it is missing. $p_{present}=0$ would give us a faster detection of present APs. However, we decided to arbitrary set $p_{present}=0.1$, meaning that we expect at least 10 percent of *N* readings for which AP *s* is present. This is to avoid the situation where APs are reintroduced to the model when they are unstable or have changed their location. Once again it can be tuned after introducing the localisation system. The parameter $p_{present}$ will play a key role when an AP has changed its location. However, that issue exceeds a scope of this work.

We could adopt a much simpler approach—we could wait for a while and if there is no signal from *s*, we detect it as missing. However, it may easily happen that most if not all the readings are gathered in the areas of the building where *s* is not detected. For example, quite often public events take place in MIS during weekends. In such case, typically only the first two floors are occupied (there are big halls and lecture rooms there) while the upper three floors are empty. The naive approach would result in removing from the localisation model most of the APs from the last three floors even though they are working. We investigate this scenario in Section 6.2 (Scenario 5). The presented case explains why the problem is more complicated and why we should look for readings for which a signal from *s* is predicted.

### 5.4. Computation Time

In the presented solution, signals from APs must be analysed to detect missing ones. Therefore a malfunction detection must be created for each AP. To reduce the costs of the system, it is recommended to reduce the number of observed APs.

**Figure 10:**
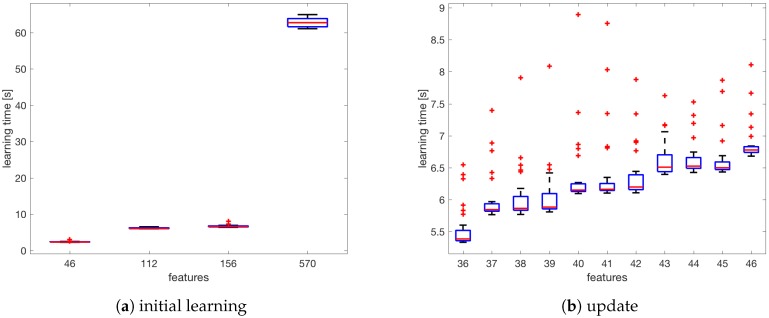
Learning time calculated for a various number of features.

Figure 10 shows the performance tests conducted on a computer with a 2.9 GHz Intel Core i5 processor and a 16 GB 1867 MHz DDR3 memory. The estimation was made on the basis of 30 tests for each set of features.

Figure 10a compares the learning time for the discussed learning sets. The presented time is the total time needed to create the localisation model ${\hat{L}}_{RF}$. The mean time varies from over one minute to less than five seconds depending on the number of features.

Figure 10b compares the learning time in the case when the localisation algorithm must be updated because of the detection of a missing AP. As the initial number of observed APs the number of APs from the infrastructure was taken. The obtained results show that the algorithm can be applied as a real-time algorithm for pedestrian navigation because the mean time lapse for creating a new localisation model after removing the missing AP does not exceed 7 s.

An additional aspect is the time necessary to collect information on RSS by a mobile device. For a device with an Android system, this time varies from 0.5 to 15 s depending on the device’s settings. However, the time obtained during the data collection was 1.5 s.

## 6. Tests and Results

### 6.1. Testing Procedure

Based on the data described in Section 4, we propose a testing procedure that will simulate the real-life situation. To do that we create a procedure *CROWD* that randomly places *K* people and their destinations. More precisely, we select randomly *K* pairs of fingerprints from the testing data set $S_{T}$ and join their locations by the shortest path consisting of elements of $S_{T}$. The shortest path is created based on the graph $G_{T}=\left(V_{T},E_{T}\right)$ connecting the fingerprint grid points as described in Section 4. Algorithm 2 is a formal description of this procedure. 

**Algorithm 2:** CROWD algorithm

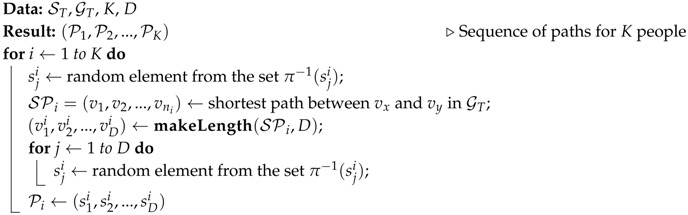



The function makeLength takes as the input sequence ${\left\{a_{i}\right\}}_{i\leq n}$ and a natural number *D*. The output is a sequence ${\left\{b_{i}\right\}}_{i\leq D}$ of length *D* such that:

(i) $\left(b_{1},b_{2},\ldots ,b_{T}\right)=\left(a_{1},a_{2},a_{3},\ldots ,a_{D}\right)$ if D≤n.
(ii) $\left(b_{1},b_{2},\ldots ,b_{D}\right)=\left(a_{1},a_{1},\ldots ,a_{1},a_{2},a_{3},\ldots ,a_{n-1},a_{n},a_{n},\ldots ,a_{n}\right)$ if D>n.

When the length of the input sequence is shorter than *D*, we repeat the first and the last element of the input sequence to make an output sequence of length *D*. We randomly decide how many times we repeat the first and the last element i.e., we randomly choose a natural number *k* between 1 and D−n so the first *k* elements are $a_{1}$, the last elements are $a_{n}$ and that the total length of the output is *D*.

In this way, we assume that each person has moved from one point to another inside the building and after or before the movement he/she spent some time at the ends of the path of his/her movement so that we have precisely *D* RSS measurements. We do not complicate the situation by investigating more complicated movements as this should suffice to consider the proposed system for an application. Real-life tests could then reveal further properties or problems. In the next section, we will show how the average error changes within the time when people are moving inside the building and how the system of detecting missing APs improves the accuracy.

In our analysis we use the time unit which is equivalent to the time between two consecutive RSS measurements. For the data sets considered in this paper this time interval is 1.5 s. We also make an assumption that each person moves by one edge of the graph $G_{T}$ between two consecutive RSS measurements. Lengths of the edges were 1 or 1.5 m. It will give us an idea how fast the missing (or present) APs are detected.

Using the *CROWD* procedure we select several paths corresponding to the movements of *K* people $\left(P_{1},P_{2},\ldots ,P_{K}\right)$ each of length *D*. Then we select five or ten APs (we check several scenarios) that are turned off and we check the effectiveness of Algorithm 1 (detection algorithm DetectAP). At every moment t≤D (t∈N) we consider two numbers: the number of correctly detected missing APs and the number of incorrectly detected missing APs. Then we add some of the missing APs and see how the system detects them at every moment.

In the next step we investigate the localisation system by looking at its final result which is a predicted location. More precisely, at every moment t≤D (t∈N) we consider the mean horizontal error for all *K* people at the moment *t* as well as the floor’s classification error. Using the notation from Section 3 we will consider for every t≤D the values $\mu E_{h}\left(\hat{L},\left\{s_{t}^{1},s_{t}^{2},\ldots ,s_{t}^{K}\right\}\right)$ and $\varepsilon_{f}\left(\hat{L},\left\{s_{t}^{1},s_{t}^{2},\ldots ,s_{t}^{K}\right\}\right)$ where the testing set $\left\{s_{t}^{1},s_{t}^{2},\ldots ,s_{t}^{K}\right\}$ are fingerprints taken at the moment *t* by all *K* people (see Algorithm 2). We compare two localisation models: ${\hat{L}}_{RF}$, the model built using all the APs without any update, and ${\hat{L}}_{RF}^{\prime }$, which is the model frequently updated (an update is carried out for every t≤D) as described in Section 5.

In the next section, we consider two occupancy scenarios (K=50 or K=200) that are characteristic for MIS building.

### 6.2. Testing Scenarios

In the further analysis, only the academic Wi-Fi infrastructure is taken into account. This is the case when there is a guarantee that there are no changes in the infrastructure of APs (location of APs, device changes and others) while the measurement series $S_{L}$ and $S_{T}$ were gathered. At this point, it is worth mentioning that the academic network inside the building consists of 46 APs whose range cover the whole building. Hence, following the notation from Definition 1 n=♯AP=46 for MIS building.

In our tests we turned off up to ten APs with the highest importance as described in Section 5.1.1 according to one of the following scenarios:

1. 5 APs were selected and turned off.
2. 5 APs were selected and turned off. However, the detection was started assuming that some other 5 APs were turned off and then turned on again. In this way we could see how fast we were able to detect that they were present.
3. 10 APs were selected and turned off.
4. 10 APs were selected and turned off. However, the detection was started assuming that some other 10 APs were turned off and then turned on again. In this way we could see how fast we were able to detect that they were present.
5. No APs were turned off. However, people were moving only on the first and the ground floor.

We tested all the above scenarios under the assumption that 200 people were moving inside the building. This figure is an estimated occupancy of the building during working hours. For scenarios 1 and 2 we also considered the case of minimal occupancy i.e., there are 30 people inside.

Our tests depended upon the parameters of Algorithm 1 (DetectAP) and the number of the testing paths:

- *N*–number of readings that need to be gathered to decide if a particular AP is missing or present. The number is the same for every AP *s*. However, we gathered only those readings for which the predictor ${\hat{f}}_{s}$ predicts that there is a signal from AP *s*.
- *K*–number of people moving on paths created using CROWD procedure (Algorithm 2).
- $p_{missing}\in \left[0,1\right]$–the decision parameter. The higher the parameter, the more likely APs are excluded from the set $S_{present}$ of APs that are used for localisation.
- $p_{present}\in \left[0,1\right]$–the decision parameter. The lower the parameter, the more likely APs are included in $S_{present}$ of APs that are used for localisation.

Firstly, we tested the algorithm when there are K=200 people inside the building (this is the average occupancy of the MIS building).

#### 6.2.1. Scenario 1

Let us look at the situation where exactly the 5 most important APs are turned off.

**Figure 11:**
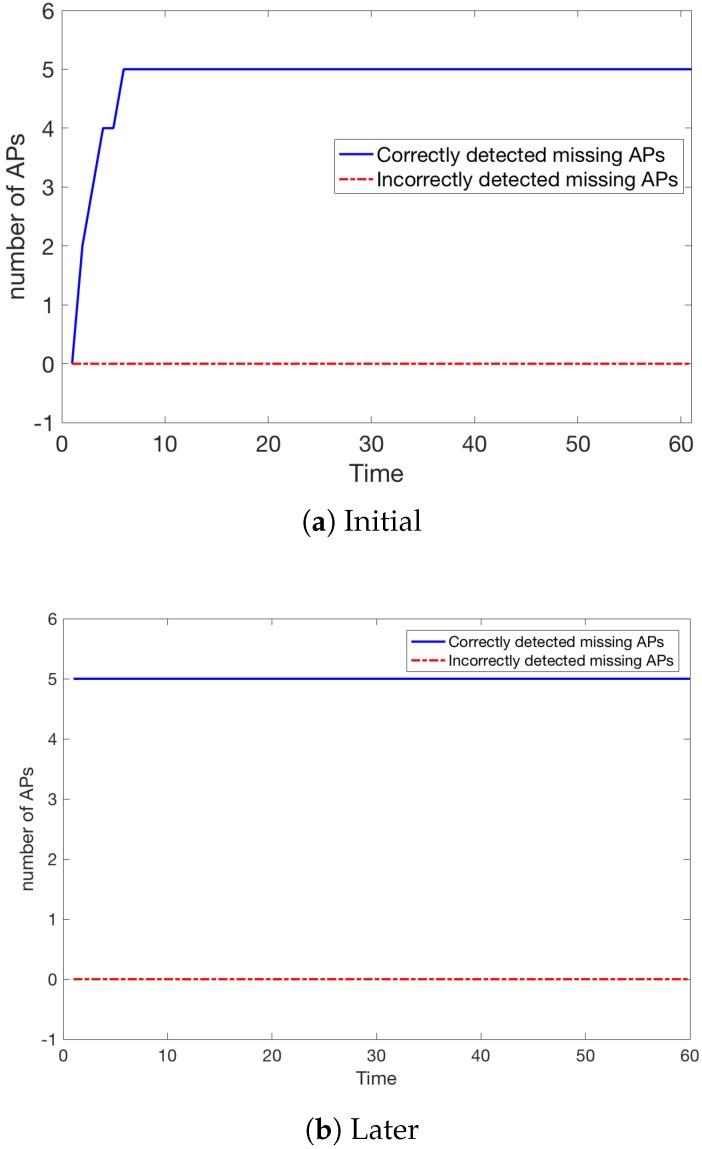
Number of missing APs detected (5 APs are not working).

It can be seen, that all missing APs were detected after time t=7 (Figure 11), giving as estimation of 10.5 s taking into account that one unit of *t* is approximately equivalent to 1.5 s.

Figure 11b shows the situation after some time when the missing APs are known from the start. Here we also assumed that there are 200 people inside the building. However, we applied the procedure CROWD once more to select a new sequence of paths. We can see that the situation is perfectly stable and no extra APs are detected as missing.

**Figure 12:**
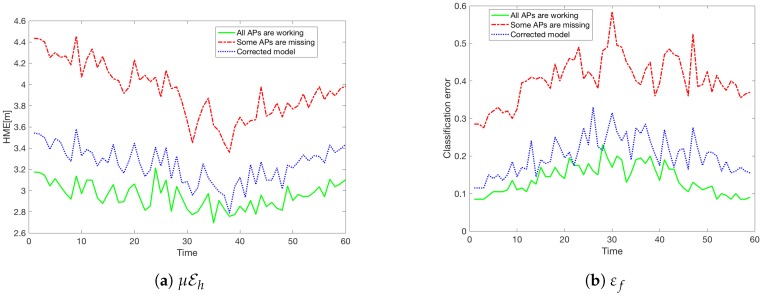
The results after the model was updated (K=200).

Hence, once the model was rebuilt by excluding the detected five missing APs, we obtained substantial gains as can be seen in Figure 12.

Let us recall that Figure 12 (as we described in Section 6.1) shows the Classification error $\varepsilon_{f}$ and the mean horizontal error $\mu E_{h}$ at every moment *t* for all K=200 people.

#### 6.2.2. Scenario 2

In this scenario, we assumed that detection is initialised with 5 APs recognised as missing although they are present. These APs are different than true missing APs which are the same as in Scenario 1. Moreover, the paths of 200 people are also the same as in Scenario 1.

**Figure 13:**
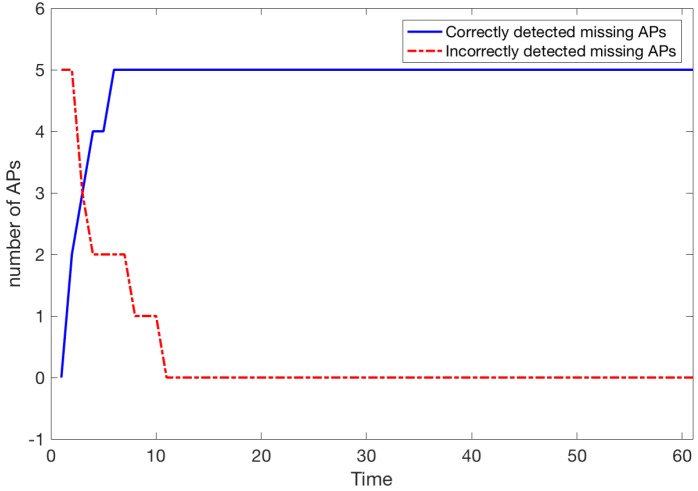
Number of missing APs detected (5 APs are not working and 5 APs are turned on again).

The moment of detection of true missing APs is the same as in Scenario 1 while the detection of 5 APs that are working again is completed by time t=11 (approx 17 s, see Figure 13). It should be pointed out that the detection of present APs could be much faster when we put $p_{present}=0$ i.e., we treat an AP as present whenever we record any signal from it (where expected). However, we suggest that the parameter $p_{present}$ should be set reasonably high to avoid APs whose signal is unstable for creating a localisation model. It should be noted that by playing with parameter $p_{present}$ we may detect an AP *s* that has changed its position or even the signal strength, as both situations usually affect its range and hence the ratio of detections of *s* where $\hat{f_{s}}$ predicts its presence. In this paper, we only simulate network malfunctions where APs are turned off. When tested later by choosing randomly (CROWD procedure) a new set of 200 paths the set of missing APs is stable and perfectly correct over time, as in Scenario 1.

#### 6.2.3. Scenario 3

**Figure 14:**
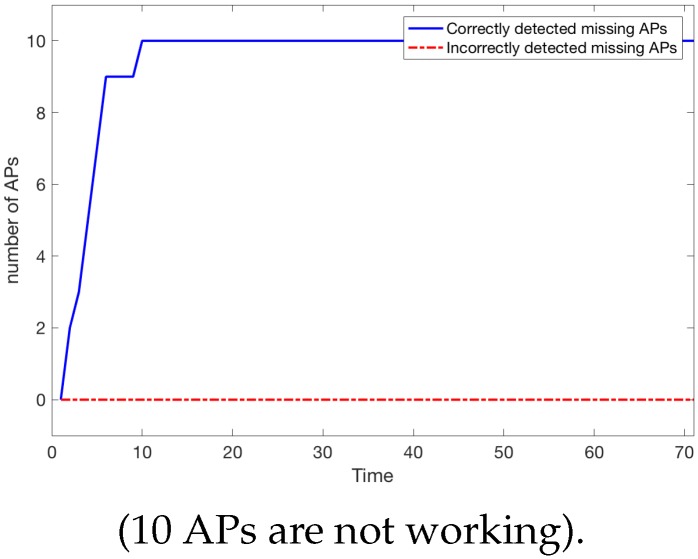
Number of missing APs detected.

Let us consider a scenario similar to Scenario 1 when the 10 most important APs were removed. Figure 14 shows that detection is almost as fast as in the case when 5 APs are removed. More precisely, after time t=12 (approx 18 s) all 10 missing APs have been detected and the situation remains unchanged. There are also no incorrectly detected missing APs.

**Figure 15:**
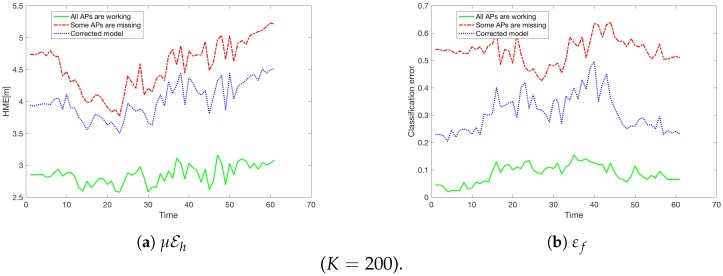
The results after the model was updated.

When the system was tested a moment later (new paths were generated using CROWD), the set of APs detected as missing remained the same. Let us see how the detection of missing APs improves the localisation:

One can see from Figure 15 that although not all the missing APs were detected, the gains obtained after the localisation model was updated are substantial.

#### 6.2.4. Scenario 4

**Figure 16:**
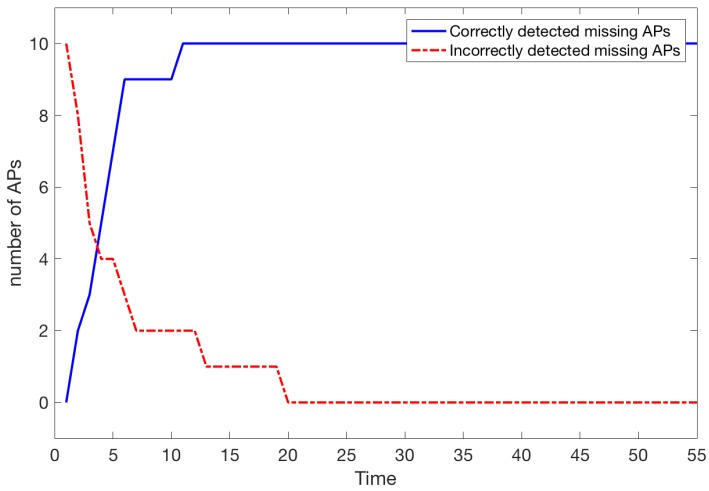
Number of missing APs detected (10 APs are not working and 10 APs are turned on again).

Here we consider a scenario similar to Scenario 2. Ten APs are turned off (the same as in Scenario 3) and additionally 10 APs are chosen that are initially treated by the localisation system as missing although they are present. The situation is very similar to the one in Scenario 3 i.e., the same APs as in Scenario 3 are recognised as missing. The system correctly detects all 10 APs that are present, although they were previously treated as missing (see Figure 16). Obviously, it takes slightly more time for the system to detect all the APs that were just turned on, compared to the situation in Scenario 2, where we had only 5 APs that had to be detected as present. The reason for that is that the input data for ${\left\{{\hat{f}}_{s}\right\}}_{s\in \mathrm{AP}}$ miss the signal strength readings from the 10 truly missing APs.

#### 6.2.5. Scenario 5

For this scenario we consider a typical situation that occurs in the MIS building, when the office area (floors 2, 3 and 4) are empty and an event takes place on the ground and first floors. We assume that 200 people(terminals) are inside the building moving only there. The naive approach, when APs are turned off whenever the server does not have any signal reported, would leave us with 12 APs removed from the model, although they are working. These are APs that are in the upper floors and their range does not cover any parts of the first and the ground floor. Removing them from localisation model would result in massive localisation error when moving in the upper floors. However, when our me a method is applied none of the APs are removed. This shows why the problem of detecting missing APs is not obvious and that the proposed method solves it.

#### 6.2.6. Scenario 1 and 2 for 30 People Moving Inside the Building

**Figure 17:**
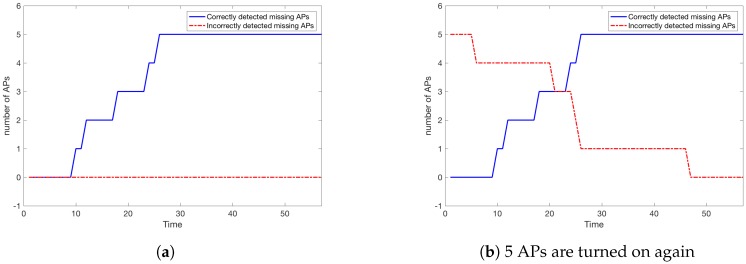
Number of missing APs detected (K=30 and 5 APs are not working).

Let us consider now Scenario 1 and 2 for a smaller number of people inside the building, i.e., K=30. In Figure 17 we see that the situation is similar to Scenario 1 and 2 with 200 people inside the building. The only difference is that missing and present APs are detected later. The time needed to correctly detect all changes is below t=30 which is approximately 45 s. This should be expected, as it takes more time for the server to gather N=100 readings for each s∈AP where the signal from *s* is expected ($\hat{f_{s}}=1$) when there are 30 people inside MIS compared to 200. Similarly to Scenario 1 and 2, the situation remains stable (the same correct APs are detected as missing from the beginning) when we test the system once again later (using the CROWD procedure).

Finally, let us explain that the above examples are truly representative. When repeating the experiments (selecting a new path using CROWD) for every scenario, we obtained similar results.

### 6.3. Comparison with Other Approach

Works [25,26,27] proposed estimation of the missing RSS values from the radio map created in the fingerprinting process. That approach works fine if each AP covers the whole analysed area but it is not possible in the case of the whole multi-floor building. However, the technique can be compared with our approach on a selected subset from our data set.

To compare the two approaches we created a testbed limited to all measures from a single floor. The learning data set consisted of 16,200 observations and the testing set 18,120 observations from the second floor. Both sets were described by signals from 40 APs observed on that floor. To create the learning set for the referential method we calculated the mean RSS for each AP separately. In this calculation, the ∅ values were not taken into consideration. The calculated mean values replaced all the ∅ values in the learning set.

**Figure 18:**
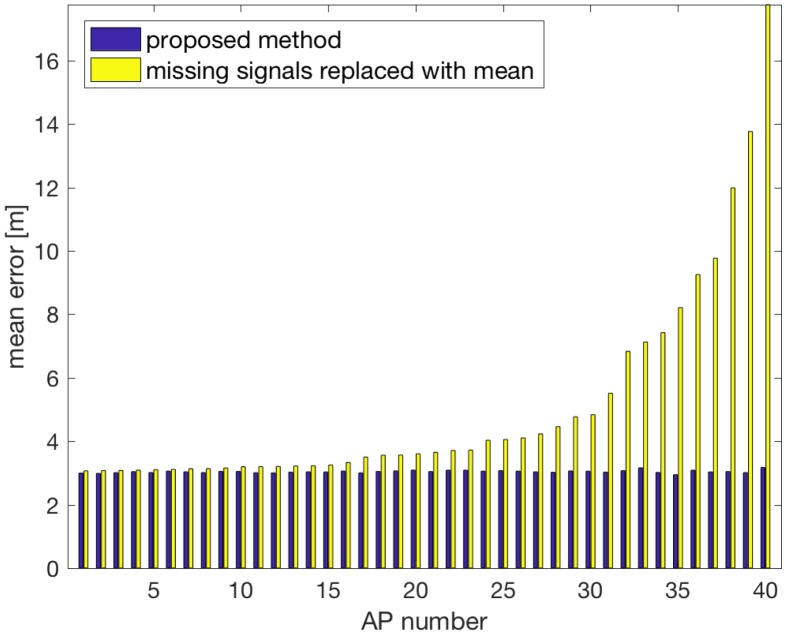
Comparison of the proposed system with replacement of missing signals with means calculated for the missing AP.

Figure 18 compares the results obtained by our system with the method proposed in [27], which replaces the missing AP signals with the mean calculated for the missing AP. The tests were conducted for each AP separately to simulate that the given AP was turned off. In the first approach, the system was updated and trained on the remaining APs. In the second approach, the signal from the turned off AP was replaced by the mean calculated for the given AP signals at all measurement points.

The results show that our method brings better results. The average difference between the methods exceeds 2 m with the maximal difference over 14 m. Therefore, we can state that the results obtained by our method are better in comparison to the replacement of the missing signals by the mean.

## 7. Conclusions and Future Works

We have proposed a complete localisation system (see Figure 5). It consists of two main components, an APs detection algorithm (Algorithm 1–DetectAP) and a localisation model created using selected APs. The DetectAP algorithm detects missing APs and allows us to rebuild the localisation model (${\hat{L}}_{RF}$).

We have showed that a reduction of the AP from the initial number of 570 sources to 76 selected ones is possible without loss of localisation accuracy. The proposed solution reduces the growth of the classification error for floor’s prediction to 0.04 and the mean horizontal error to 2 m in comparison to 0.3 and 7.5 m for the estimation made by non-updated model. That gives a reduction of the mean horizontal error by 5.5 m and the classification error for the floor’s prediction by 0.26 (see results for $\varepsilon_{f}$ and $\mu E_{h}$ respectively in Table 2).

We analysed the DetectAP algorithm by simulating real-life situations when *K* people are moving inside the building (K=30 or K=200). We investigated the situation of turning off either 5 or 10 APs. The system correctly detected missing APs in both cases. The time needed.

The presented system can be used to improve existing localisation systems based on multi-source signals. It also has practical applications. The localisation with a few meters accuracy is useful and can be applied to monitor mobile medical facilities at hospitals; to track products on the shop floor for example in a bus factory; to localise supervised residents in a health centre.

The detection algorithm is built in such a way that it may detect not only the fact that an AP is turned off. When we move an AP in a completely different location, it will also most probably be detected as missing. This is due to the fact that DetectAP searches for signals where they are expected. However, this property of the proposed detection algorithm is not analysed in this paper and will be a subject for further research by the authors.

## Figures and Tables

**Table 1:** Number of fingerprints in each series for MIS building.

S	$S_{L}$	$S_{T}$
♯S	43680	46760

**Table 2:** Results of the localisation for removed APs: before and after update.

Removed	$\mu E_{h}$ [m]	$\mu E_{h}$ [m]	$\varepsilon_{f}$	$\varepsilon_{f}$
APs	before	after	before	after
0	4.47	4.47	0.93	0.93
1	4.74	4.47	0.91	0.93
2	5.93	4.72	0.85	0.92
3	6.77	4.94	0.81	0.92
4	7.36	5.07	0.78	0.92
5	7.80	5.17	0.73	0.92
6	8.09	5.34	0.71	0.91
7	9.36	5.50	0.69	0.90
8	10.66	5.98	0.68	0.90
9	11.13	6.12	0.65	0.89
10	11.95	6.41	0.63	0.89
